# Reliability of the isometric mid-thigh pull peak force in Irish schoolboy rugby players

**DOI:** 10.17159/2078-516X/2021/v33i1a9433

**Published:** 2021-09-09

**Authors:** D O’Dowd Hill, CA Lodge, DT Browne

**Affiliations:** HealthCORE, Institute of Technology Carlow, Carlow, Ireland

**Keywords:** team sports, IMTP, youth, maximal strength

## Abstract

**Background:**

Maximal strength is a key variable within youth rugby union and is therefore of merit when testing and monitoring youth rugby athletes. Isometric mid-thigh pull peak force (IMTP PF) has been observed to be a reliable, valid, and safe means of assessing maximal strength in previously researched cohorts. Currently, there exists a distinct lack of literature with regards to the use of IMTP PF with non-elite youth athletes.

**Objectives:**

(1) Investigate the intra-day reliability of the IMTP PF with respect to the three age-grades of Irish schoolboy rugby union, (2) investigate the inter-day reliability of the IMTP PF within Irish schoolboy rugby union.

**Methods:**

The current study utilised self-selected body positions of 84 non-elite schoolboy rugby union athletes (age: 14.7 ±1.7 years; height: 170.6 ±10.3 cm; mass: 63.9 ±14.8 kg).

**Results:**

IMTP PF was observed to be a reliable measure of peak force both inter- and intra-day (Intra-day: Coefficient of Variation 3.3%; Interclass Correlation Coefficient 0.99; 95% Confidence Interval 0.99 – 1.00; Inter-day: Coefficient of Variation = 5.1%; Interclass Correlation Coefficient 0.99; 95% Confidence Interval 0.98 – 1.00). A change of 10.9 kg should be deemed worthwhile when working with Irish schoolboy rugby players.

**Conclusion:**

The IMTP PF is a safe and reliable measure of maximal strength in non-elite youth rugby athletes.

Rugby union is an intermittent collision sport, in which maximal muscular strength is of importance.^[1]^ This importance is due to the relationships between increased maximal strength and improvements in both dynamic performance and rugby specific skills, such as jumping, sprinting, scrummaging, tackle success rates, and line break rates.^[1, 2]^ Increased maximal strength also has the ability to reduce injury rates and improve psychosocial wellbeing in athletes.^[2, 3]^ The importance of maximal strength in rugby union is not limited to the adult game (>U20). This is apparent when you consider that maximal strength is a distinguishing factor between playing positions, age grades and playing levels in youth rugby union.^[4, 5]^ Therefore, one must assume that the monitoring and testing of maximal strength should be a priority when dealing with youth rugby players across the development pathway.

Maximal strength can be defined as the ability to exert maximal force against an external resistance and requires a maximal voluntary contraction.^[6]^ Within youth sports, this has previously been measured through the use of repetition maximum testing, during which a participant is instructed to complete multiple sets of increasing weight for a prescribed number of repetitions per set until failure.^[7]^ More recently, portable force plates and specialist isometric racks have become cheaper and more accessible. This has allowed isometric maximal strength testing to become more common in both practical and academic settings.^[8]^ This is also due to the proposed benefits of increased safety, lack of familiarisation needed, decreased fatigue and high reliability when compared to the more conventional resistance-based lifts, such as, squat, bench press and deadlift.^[9–11]^

The isometric mid-thigh pull (IMTP) is an isometric means of testing maximal strength which has previously been shown to be reliable (Interclass Correlation Coefficient (ICC) >0.90, Coefficient of Variation (CV%) <10%) and valid (1RM Squat, r=0.86; Counter Movement Jump (CMJ), r=0.82) irrespective of previous cohorts used.^[11]^ To date, no studies have investigated the reliability of the IMTP PF within grassroots schoolboy rugby union. Previous studies investigating IMTP PF in rugby union have taken place with older elite cohorts (u16+). ^[4, 12]^ The IMTP is designed to mimic the second pull of the Clean, which is the phase which generates the highest peak force during the movement.^[13]^ There are strong associations between IMTP peak force (IMTP PF) and other dynamic resistance-based movements, such as the Olympic lifts, squats and deadlifts.^[9, 11, 14]^ Further evidence of the utility of the IMTP PF to rugby is apparent when it’s associations with improved dynamic movements and rugby-specific skills are considered.^[1, 11, 15]^ Although most of these studies are limited to adult cohorts, there also exists known links between the IMTP PF and dynamic performance for youth, indicating its relevance irrespective of age.^[15]^

The current study aims to investigate the reliability of the IMTP PF with respect to Irish schoolboy rugby union. The objectives undertaken by this study were to (1) Investigate the intra-day reliability of the IMTP PF with respect to the three age-grades of Irish schoolboy rugby union (2) investigate the inter-day reliability of the IMTP PF within Irish schoolboy rugby union.

## Methods

### Study design

The current study used an observational cross-sectional design. All male rugby players within an Irish secondary school, participating in the Connacht schools U14, Junior and Senior competitions were invited to participate. Consent was sought from a legal guardian following an open invitation information night. All participants were required to attend the after-school gym session which testing took place. Anthropometric measurements of height (cm) and mass (kg) were investigated across the chronological age grades. Isometric mid-thigh pull reliability (ICC & CV%) was investigated intra- and inter-day, with inter-day testing being undertaken by a representative cohort one week after intra-day testing.

### Participants

Eighty-four participants from the three age-grades (U14; Junior, U16; Senior, U19) elected to take part in the study and met all the inclusion criteria. Ethical approval was granted by the relevant ethics board in accordance with the Declaration of Helsinki. The participant’s mean chronological age, height and mass were age, 14.7 ±1.7 years; height, 170.6 ±10.3 cm; mass, 63.9 ±14.8 kg. Of the 84 participants, 10 participants were retested to allow for inter-day reliability testing (Table 1).

### Procedures

**Fig. 1:**
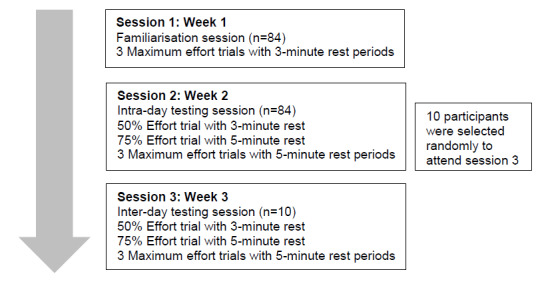
The protocol for the warm-up and testing trials.

Participants wore normal gym clothing during testing. Immediately upon entering the gym, body mass was measured using a portable scale (Seca, Germany). Standing and seated height were then measured using a portable stadiometer (Seca, Germany). Following the anthropometric measurements, a standardised 10 minute dynamic warm-up was completed. Isometric mid-thigh pull (IMTP) was then measured using a portable force plate (Hurlabs, Finland). A protocol consisting of three sessions, each one week apart was utilised (Figure 1). The first session was a familiarisation session, which consisted of three maximal efforts of the IMTP with a three-minute rest period between trials. The second session was the first full testing session and was used to test intra-day reliability. The third session was the second full testing session and was used to test inter-day reliability. During the full testing sessions, two warm-up repetitions were completed at 50% and 75% self-rated intensity respectively, with a three-minute rest period between each warm-up trial. All participants then completed three trials at 100% intensity, with a five minute rest period between each of the three trials. The protocol for the warm-up and testing trials is outlined below (Fig. 1).

### Isometric mid-thigh pull peak force (IMTP PF)

IMTP PF has previously been reported as reliable (CV≤4.3%, ICC>.92), valid and responsive to resistance training, irrespective of previously tested cohorts.^[11]^ All IMTP PF data were collected at 1 000 Hz using a portable force plate (Force Platform FP8 2003, Hurlabs, Finland), which was placed under a fixed bar. All data were extracted using the corresponding software suite provided with the force plate (Hurlabs Force Platform Software Suite 2.65.5.6, Hurlabs, Finland). The force plate was zeroed between all participants. During testing, the participants were asked to self-select a body position from which they ‘felt comfortable to pull as hard as possible’, with the bar being in a fixed position at ‘mid-thigh’ (halfway between the patella and iliac crest). The use of a self-selected body position during isometric mid-thigh pull peak force testing has been shown to be reliable (ICC=.99), as well as having a small effect (Cohen *d*=.037) when compared to eight other body positions.^[10]^ Bar height was visually verified as being acceptable by the lead supervisor. Minimal pre-tension was allowed to ensure that there was no slack in the body prior to the initiation of the pull. The participant was then instructed to ‘pull as hard and fast as possible’ for five seconds once the verbal cue of ‘Go!’ was given by the lead examiner. During the trial, the lead examiner verbally counted down the trial time aloud; ‘five, four, three, two, one’, before giving acknowledgment of completion of the trial by shouting ‘Stop’.

### Statistical analysis

All descriptive statistics were computed by SPSS (IBM, V.25, 2017) and Microsoft Excel (Microsoft, 2010) from the data set. All variables were calculated for the overall cohort and with respect to the age-grades. Through the use of a Kolmogorov-Smirnov test the data was deemed to be normally distributed. Intra- and inter-day random variability was determined using mean coefficients of variation (CV%) and intra-class correlation coefficients (ICCs) (2,1) to determine both absolute and relative reliability. Acceptable thresholds were determined using a CV of ≤ 10%.^[16, 17]^ Ninety-five percent confidence intervals were calculated for all variables. Magnitudes of ICC were classified according to the following thresholds: 0.9 nearly perfect; 0.7–0.9, very large; 0.5–0.7, large; 0.3–0.5, moderate; and 0.1–0.3, small.^[16]^ Standard error of the mean (SEM) and smallest worthwhile change (SWC) were also calculated to allow for inferences to be made about the variability and practical application of the IMTP PF.

## Results

### Intra-day reliability (Table 2 and Fig. 2)

The IMTP PF exhibited a nearly perfect ICC across all age-grades for intra-day testing (ICC ≥ 0.97; 95% CI ≥ 0.94 - ≥ 0.98). The CV% was also below the threshold of acceptability for all age-grades (CV% ≤ 3.7%). The IMTP PF had a SEM of 5.9 kg and a SWC of 10.9kg.

### Inter-day reliability (Table 3 and Fig. 3)

The IMTP PF exhibited a nearly perfect ICC across the two testing sessions (%; ICC 0.99; 95% CI 0.98 – 1.00). The CV% was also below the threshold of acceptability for all age-grades (CV% = 5.1%).

## Discussion

The current study aimed to investigate the reliability of the IMTP PF within Irish schoolboy rugby union. While using a self-selected body position, with one familiarisation session, the IMTP PF was reliable both intra- and inter-day with respect to the Irish schoolboy rugby union.

### Reliability

With respect to intra-day reliability, the IMTP PF for the overall cohort had nearly perfect ICC values (ICC 0.99; 95% CI 0.99 – 1.00) and had a CV% well below the acceptable threshold (3.3%). This indicates that the IMTP PF is a reliable measure when considering the maximal isometric strength of schoolboy rugby players. This is in agreement with previous studies on youth rugby union players, which observed the ICC and CV to be 0.97 and 3.5% respectively.^[4]^ Although, this study was carried out on elite youth from U16, U18, and U21, it utilised prescribed hip and knee angles. It is therefore promising that the reliability measures of the IMTP PF were improved upon slightly when using self-selected knee and hip angles within a younger, non-elite cohort of rugby union players.

When compared to intra-day, the inter-day reliability of IMTP PF maintained its nearly perfect ICC values (ICC 0.99; 95% CI 0.98 – 1.00), while CV% increased to 5.1%. Although there are no studies assessing the inter-day reliability of the IMTP PF in youth rugby players, the current study is in contrast with a study conducted on elite youth footballers with an age of 18.4 years±2 years.^[18]^ Musham’s study took four testing sessions to achieve comparable reliability (CV% = 5.8%; ICC 0.88; 95% CI 0.77 – 0.94). Although comparisons with this study must be done cautiously as limitations may be apparent. During Musham’s study, a ‘minimum of two trials’ was completed by each participant interspersed by 30-second rest periods between each maximum effort trial. Due to this lack of recovery, fatigue may be a confounding factor when trying to measure reliability for isometric strength protocols.^[19]^ It is therefore surprising that the recommendations arising from Musham’s study are that a minimum of four sessions comprising of six trials per session with five minute rest periods are needed to achieve adequate reliability when working with novice athletes.^[18]^

### Age grade

The current study was the first to assess the reliability of individual age grades with respect to the IMTP PF. The reliability was high across all three age grades. The junior cohort had the highest reliability (CV% = 3.0%; ICC, 95% CI; 0.99, 0.98 – 0.99), followed by seniors (CV% = 3.1%; ICC 0.97; 95% CI 0.94 – 0.99), and U14s (CV% = 3.7%; ICC 0.97; 95% CI 0.94 – 0.98). Both the reliability of the three groups and trend of the junior group being of the highest reliability are in contrast with inferences made in previous studies which cited training age as a confounding factor in the reliability of the IMTP PF in youth athletes.^[16, 18]^

### Limitations

The current study only assessed inter-day reliability across two days, with a relatively small cohort. This may limit the ability to test systematic bias across multiple testing dates. The lack of inclusion of U14 participants within the inter-day testing may also be a limitation.

### Practical application

With regard to schoolboy rugby players, the IMTP PF is a highly reliable measure of maximal strength irrespective of chronological age. This is true for both inter- and intra-day. The use of a single familiarisation session and self-selected body position is sufficient with respect to reliability and may therefore be utilised when assessing youth athletes. The current study’s results also indicate that a single trial may also be sufficient when measuring peak force. A change of 10.9kg should be deemed worthwhile when working with Irish schoolboy rugby players. The adoption of these methods may allow for practitioners to test and monitor large groups of youth rugby athletes in a time-efficient and safe manner.

## Conclusion

A single trial of the isometric mid-thigh pull with a self-selected body position is a reliable measure of both intra- and inter-day maximal strength in non-elite schoolboy rugby athletes.

## Figures and Tables

**Fig. 2:**
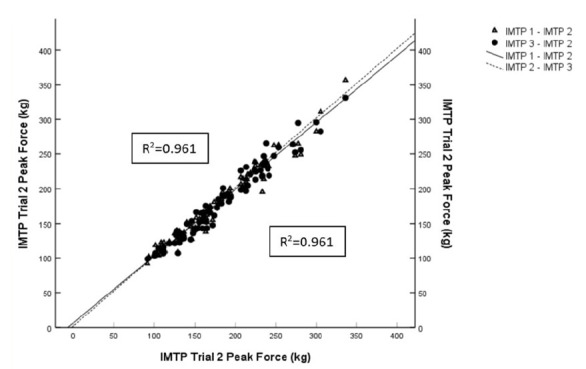
Comparative intra-day scatterplot for the whole cohort (n=84) with lines of best fit.

**Fig. 3:**
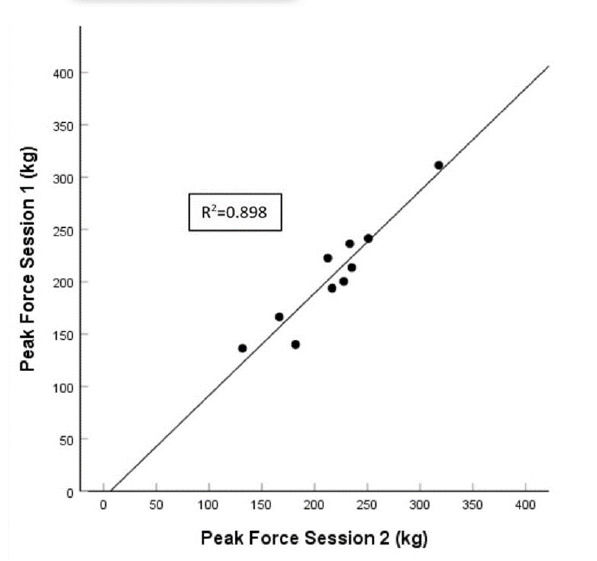
Comparative inter-day scatterplot for the representative cohort (n=10) with lines of best fit.

**Table 1:** Analysis of cohort

	Intra-day (n)	Inter-day (n)
**Overall**	84	10
**U14**	29	0
**Junior (U16)**	32	5
**Senior (U19)**	23	5

**Table 2:** Intra-day reliability (n=84)

	Trial 1 (kg)	Trial 2 (kg)	Trial 3 (kg)	CV%	ICC (95% CI)
**Overall (n=84)**	180.6 ± 54.3	180.5 ± 55.2	178.2 ± 54.1	3.3	0.99 (0.99 – 1.00)
**U14 (n=29)**	133.0 ± 21.9	132.9 ± 24.5	131.6 ± 22.7	3.7	0.97 (0.94 – 0.98)
**Junior (U16) (n=32)**	186.6 ± 37.6	187.0 ± 42.3	184.4 ± 42.2	3.0	0.99 (0.98 – 0.99)
**Senior (U19) (n=23)**	242.3 ± 36.7	241.4 ± 34.5	238.0 ± 33.6	3.1	0.97 (0.94 – 0.99)

Data are expressed as mean ± SD unless indicated otherwise. CV%, Coefficient of Variation; ICC, Interclass Correlation Coefficient; CI, Confidence Interval.

**Table 3:** Inter-day reliability (n=10)

	Trial 1 (kg)	Trial 2 (kg)	Trial 3 (kg)	Trial 4 (kg)	Trial 5 (kg)	Trial 6 (kg)	CV%	ICC (95% CI)
**Overall (n=10)**	203.4 ± 51.1	200.8 ± 51.7	195.4 ± 41.1	214.5 ± 52.2	202.1 ± 46.5	202.6 ± 49.1	5.1	0.99 (0.98 – 1.00)

Data are expressed as mean ± SD unless indicated otherwise. CV%, Coefficient of Variation; ICC, Interclass Correlation Coefficient; CI, Confidence Interval. Trial 1 to 3 took place on day 1, while Trial 4 to 6 took place on day 2.
